# Acute interstitial nephritis – a reappraisal and update 

**DOI:** 10.5414/CN108386

**Published:** 2014-07-30

**Authors:** Rajeev Raghavan, Garabed Eknoyan

**Affiliations:** Division of Nephrology, Department of Medicine, Baylor College of Medicine, Houston, TX, USA

**Keywords:** acute interstitial nephritis, acute tubulo-interstitial nephritis, acute kidney injury, acute renal failure, chronic kidney disease

## Abstract

Acute interstitial nephritis (AIN) is an under recognized and under diagnosed cause of acute kidney injury (AKI). It is estimated to account for 15 – 20% of cases of AKI; it is the reported diagnosis in 2.8% of all kidney biopsies, and 13.5% of biopsies done specifically for acute renal failure. Considerable evidence implicates antigen initiated cell-mediated injury in the pathogenesis of AIN. Drugs account for 70% of all cases, with over 150 different agents incriminated. The remaining cases are due to infections, autoimmune diseases, and rarely idiopathic. The central component of renal injury in AIN is altered tubular function, which usually precedes decrements in filtration rate. The key to early diagnosis is vigilance for the presence of tubular dysfunction in non-oliguric individuals, especially in patients with modest but gradual increments in creatinine level. The utility of urinary biomarkers to diagnose AIN in its early nascent and potentially reversible stage remains to be determined. Prompt recognition, elimination of the offending source of antigen, and use of a limited course of steroid therapy where indicated, will result in complete resolution in ~ 65% of cases, partial resolution in up to 20%, and irreversible damage in the rest.

## Introduction 

It is only in the past century that the functions of the kidney were defined, its varied diseases identified, and its central role in homeostasis established. Until then, and throughout most of medical history, the kidney was considered a tubular secretory organ adjunct to the gastrointestinal tract in the vital process of nutrition. After the milestone 1827 report of dropsical albuminuric medical cases by Richard Bright (1789 – 1858), the renal lesions of Bright’s disease came to be considered as inflammatory (nephritis) in nature and classified in 1858 by Rudolph Virchow (1821 – 1902) as “nephritis” affecting the tubules (“parenchymatous” nephritis), the interstitium (“interstitial” nephritis), or the vasculature (amyloid). Diseases of the kidney then came to be grouped together under the indefinite terms of “nephritis” or “Bright’s disease” and classified as either acute or chronic. It is only after improvements in the resolution power of microscopes and refinements in tissue processing that “glomerular” nephritis was added to the list of nephritides in 1869 by the Swiss pathologist Edwin Klebs (1834 – 1913) [1]. 

Early tribulations and classifications notwithstanding, most diseases of the kidney continued to be considered as tubulopathies rather than glomerulopathies through the first decades of the 20^th^ century. It is within this context that the pathologic diagnosis of “acute interstitial nephritis” (AIN) was described in 1898 by William Thomas Councilman (1854 – 1933), then pathologist-in-chief at the Brigham Hospital [2]. Notably, both Klebs and Councilman were pathologists interested in infectious diseases who made their observations at a time when cellular pathology and the role of microorganisms in disease causation were at their prime; and “scarlatina” was considered a major cause of “acute nephritis” or “acute renal dropsy” [3, 4]. Indeed, Klebs described his eponymous glomerular lesion in a patient with scarlet fever, 9 years after the first case of acute interstitial nephritis was described in 1860 by the German internist Michael Anton Biermer (1827 – 1892) in a 4-year-old child who died from scarlet fever [5]. 

**Figure 1.**
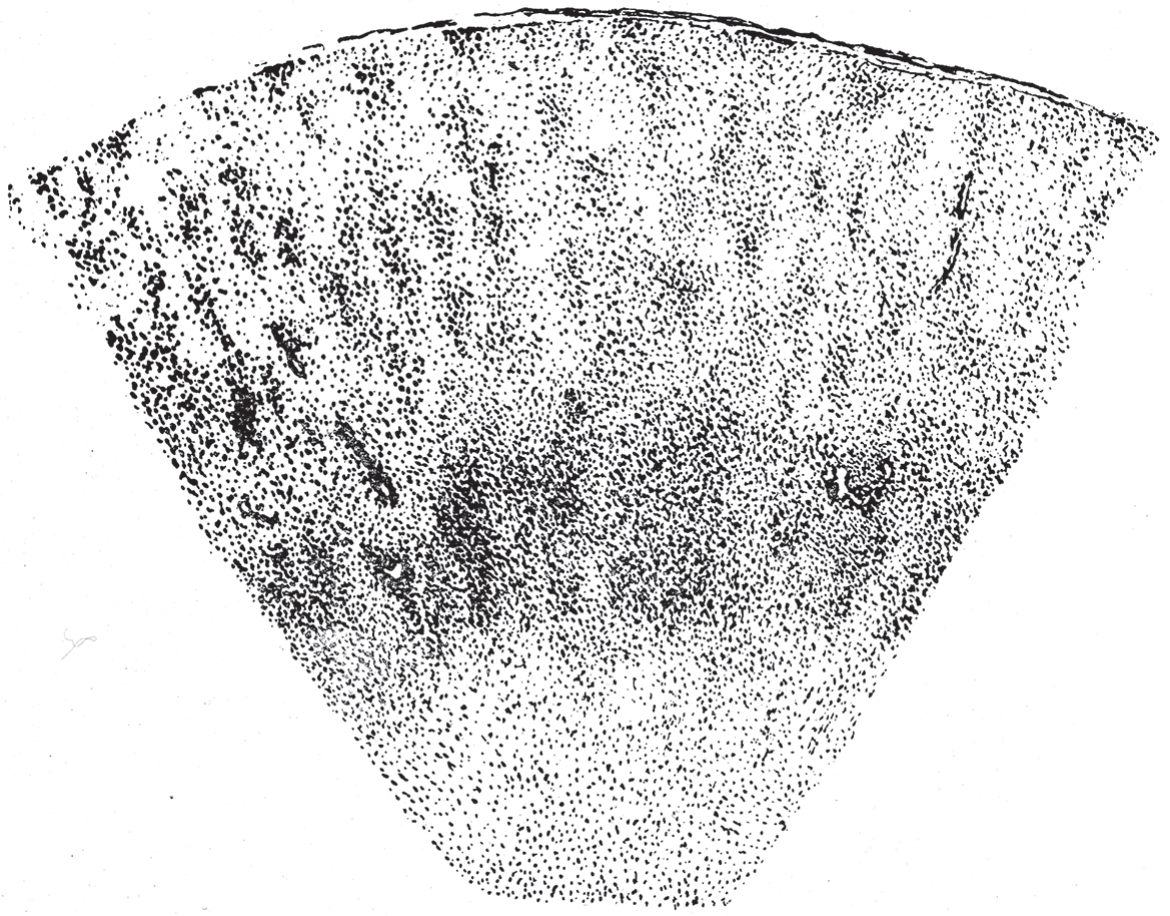
Cross section of kidney magnified 5 times to show the foci of regional distribution of cellular infiltrates in acute interstitial nephritis. (Reproduced with permission from reference number [2]).

Councilman’s now classic paper on AIN was based on a review of the literature and his own post-mortem examination of 42 cases of “non-suppurative inflammatory interstitial lesions” of the kidney occurring predominantly in patients with streptococcal infections. He described the pathology as “an acute inflammation of the kidney characterized by cellular and fluid exudation in the interstitial tissue, accompanied by, but not dependent on, degeneration of the epithelium; the exudation is not purulent in character, and the lesions may be both diffuse and focal.” He identified the infiltrating cells as “plasma cells that had migrated from the blood vessels and multiplied locally by mitotic division.” This prescient description of the mononuclear infiltrating cells is a telltale sign of the activated T cells that would be identified in the immunopathogenesis of AIN a century later. He localized the foci of cellular infiltrates to three sites: the boundary zone of the pyramids, the subcapsular region of the cortex, and around the glomeruli (Figure 1). These careful observations based on examination of the entire kidney at post-mortem are particularly relevant to appreciating the varied renal manifestations of AIN [2, 6]. 

Subsequent reports confirmed Councilman’s observation that non-suppurative lesions of the renal interstitium appeared after short (3 – 5 days) but variable periods after the onset of an acute streptococcal infection. This concept of acute tubular and interstitial injury following an acute infection was so well established that initial reports of acute tubular necrosis (ATN) were termed *acute hematogenous interstitial nephritis* [7]. In fact, on careful review of case reports of AIN published before 1941, it becomes obvious that several cases reported as AIN were actually the result of septic shock with classic lesions of ATN rather than those of AIN described by Councilman [2]. Difficulty in the differential diagnosis of these two variants of tubulo-interstitial diseases (AIN and ATN) persists to this day [6]. The clinical differentiation of ATN from AIN is confounded by the recent introduction of the inclusive term “acute kidney injury” (AKI) that has achieved widespread use generally without an attempt to identify or specify the etiology or actual pathologic cause of the acute renal injury. 

The introduction of antibiotics in the 1940s and consequent eradication of fatal streptococcal infections resulted in a near total loss of interest in the entity described by Councilman, and attention shifted to ischemic and nephrotoxic ATN as the predominant causes of acute renal failure. It is rather ironic then that interest in AIN was revived in the 1960s when the very antibiotics used to treat streptococcal infections were identified as a cause of AIN [6]. In fact, the bulk of current reports of AIN are for drug-induced AIN, and the number of drugs implicated as causing AIN continues to increase, as does that of the variation in the clinical and laboratory manifestations associated with the renal injury. Given the varied spectrum of its clinical manifestations (fever, rash, multi-organ involvement) AIN can best be considered as a clinico-pathologic syndrome that develops in diverse conditions (infections, drugs, systemic diseases, idiopathic), which is characterized by an acute deterioration of kidney function, the pathologic features of which remain those described by Councilman [2]. 

The descriptive term *acute tubulo-interstitial nephritis* (ATIN) was introduced in the 1960s in describing ATN cases and was subsequently applied to those of AIN [6]. The advantage of using *tubulo-interstitial* rather than just *interstitial* derives from the fact that although the dominant morphologic features of AIN are those evident in the interstitium, the tubules are characteristically affected, albeit to variable degrees that may be difficult to detect on light microscopy. In fact, the tubules play an important role in the immunopathogenesis of AIN, and tubular dysfunction as a rule precedes clinically evident decreases in glomerular filtration rate [6]. 

**Figure 2.**
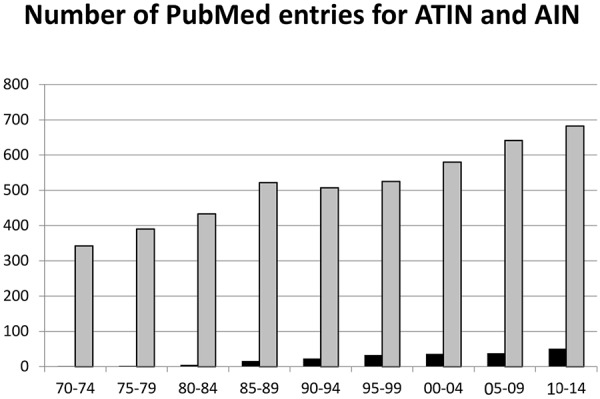
Number of reports listed on PubMed as acute tubulointerstitial nephritis (shown in black) and of acute interstitial nephritis (shown in grey) over the past 45 years. ATIN = acute tubulointerstitial nephritis; AIN = acute interstitial nephritis.

The importance of these functional and structural considerations notwithstanding, the term *tubulo-interstitial* is likely grammatically incorrect (tubulo-interstitial, tubular-interstitial) and certainly cumbersome to pronounce compared to the simpler “interstitial”. This has resulted in the broader acceptance of the term “acute interstitial nephritis” in the literature. As shown in Figure 2, a search for AIN yields over 4,800 entries, whereas that of ATIN yields a mere 207; in which 20% of the “ATIN” cases are those of tubulo-interstitial nephritis with uveitis (TINU), an acronym that has been established itself in the medical nomenclature as TINU. It may be time therefore, to adopt the simpler term of “acute interstitial nephritis” (AIN) and limit that of *tubulo-interstitial *to cases of TINU. 

## Prevalence 

The definitive diagnosis of AIN requires a kidney biopsy, as well as laboratory or clinical identification of the causative factor. As such, most estimates of the prevalence of AIN are based on retrospective reviews of biopsy registries, and the actual incidence of AIN goes grossly underestimated in part due to the relatively low index of suspicion with which the diagnosis is entertained clinically, and the general reluctance to biopsy cases of AIN, particularly when the laboratory abnormalities are minor and the symptoms subside after a change of medications [8]. 

Figures on the prevalence of AIN derived from retrospective reports of large (> 500 biopsies) kidney biopsy registries are shown in Table 1 [9, 10, 11, 12, 13, 14, 15, 16, 17, 18, 19, 20, 21, 22, 23, 24, 25]. In most published registries, glomerulonephritides are the most common diagnosis and the primary reason for kidney biopsy. The reported incidence of AIN ranges from 1% to 10% of the total biopsies examined, with an overall average of 2.8%. The incidence of AIN increases among biopsies done specifically to evaluate acute renal failure of unknown origin, where it ranges from 6.5% to 35%, with an overall average of 13.5%. These relatively wide ranges reflect the varied and often subjective reason for which kidney biopsies are performed in different regions rather than a difference in actual occurrence of AIN. 

A review of kidney biopsies from our institution during the period of 2000 – 2010 revealed 3,765 adult kidney biopsies, 240 (6.4%) of which were diagnosed as either acute or chronic TIN. Of these 240 cases, AIN represented 150 cases (62%) and chronic (including acute on chronic) interstitial nephritis (CIN) 90 cases (38%). These latter 90 cases likely reflect a late diagnosis of AIN after irreversible fibrosis had set in and emphasize the need for early diagnosis before the onset of fibrosis [6, 26]. 

Autoimmune diseases, primarily systemic lupus erythematosus, represented 46 (31%) of our AIN cases. Another 28 (20%) were drug-induced, in which antibiotics (13 cases) and NSAIDs (6 cases) were the most common implicated agents. Infection was reported as the cause of AIN in 12 (8%) cases; 6 of which were secondary to HIV, in whom only minor glomerular changes were present. In 54 cases (36%), the exact etiology of AIN was unknown. It is important to note that only 2 of our 150 AIN cases were reported in the literature reflecting the limitation of literature reviews to determine the prevalence of AIN. Additionally, the unusual finding of such a high number of autoimmune diseases in our series clearly reflects the bias of doing kidney biopsies in such cases. 

## Pathogenesis 

AIN is part of the broad spectrum of idiosyncratic delayed hypersensitivity immune reactions to foreign antigens, the defining feature of which is the associated acute renal injury [27]. Convincing evidence from animal and human studies implicate a central role to a variety of antigen-reactive T cells in the spectrum of dysregulated immunologic responses that ensues antigen exposure, including skin eruptions, eosinophilia, fever, hematologic abnormalities, and solid organ involvement (liver, lung, kidneys). The high blood flow to the kidneys, where antigens are filtered, secreted or concentrated, renders them at increased risk of exposure. The idiosyncratic nature of the reaction is clearly evidenced from the clinical features of drug-induced AIN, which: 1) occurs in only a small number of exposed individuals; 2) it is not dose-related; 3) it is associated with other systemic manifestations of hypersensitivity (fever, skin rash, eosinophilia, arthralgia); and 4) the reaction recurs on re-exposure to the same drug or one of its congeners [6]. 

**Figure 3.**
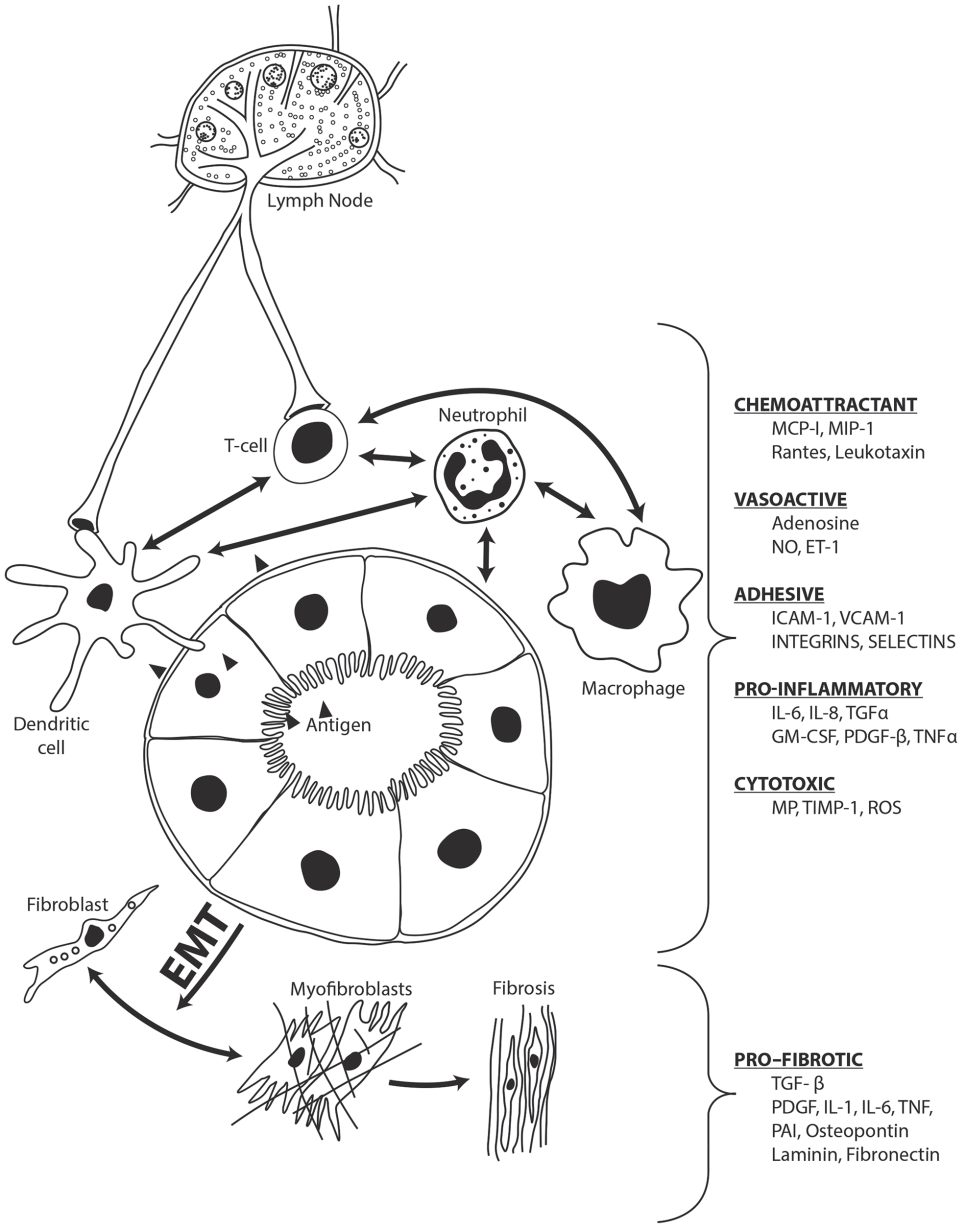
Pathogenesis of AIN. The process begins with the recognition and subsequent processing of the putative antigen by dendritic cells that endocytose, process, and express the peptides on their surface MHC II molecules, which they then present to the naïve lymphocytes in the regional lymph nodes. See text under Pathogenesis for the subsequent course of events. EMT = epithelial mesenchymal transition.

Considerable evidence accrued over the past decade implicates the underlying immunologic role of cell-mediated injury in the previously enigmatic pathogenesis of AIN, which for simplicity will be considered in three successive but overlapping phases: an *antigen recognition* and presentation phase, an *integrative* or regulatory (primarily cellular) phase, and an *effector* or mediator (primarily humoral) phase [6]. In the first phase, either the resident peritubular interstitial cells or injured tubular epithelial cells function as antigen presenters. The normal renal interstitium contains resident monocytes, previously considered to be macrophages but recently identified to be mainly dendritic cells (DC), whose long processes create an extensive, contiguous network within the renal parenchyma and come in direct contact with tubular epithelial cells (Figure 3) [28, 29, 30]. These are specialized sentinel cells of the immune system whose stella-styled projections probe their environment for what has been termed danger signals, be they foreign antigens or injured and stressed tubular epithelial cells [30, 31, 32, 33]. The normally quiescent DCs when exposed to antigens or damage signals are activated; they endocytose, process, and express the incriminated antigenic components as peptides located on their surface MHC II molecules. Once activated, DCs migrate through the renal lymphatic vessels to regional lymph nodes where they present the antigen to the residual naïve T cells, which are then activated and migrate to the antigenic source or injury emitting the danger signal (Figure 3) [34, 35, 36, 37]. DCs have been shown to take up potentially antigenic molecules, small enough to be filtered (ovalbumin) directly from the tubular lumen [29, 35]. In addition to the dendritic cells, the renal interstitium contains dormant macrophages and fibroblasts that are also activated and contribute to this initial inflammatory response, which is further magnified by recruited neutrophils, including eosinophils. Depending on the intensity and duration of exposure to the antigen, new fibroblasts are recruited from circulating bone marrow stem cells, pericytes or from epithelial and endothelial cells by mesenchymal transition (EMT) that magnify the potential for irreversible fibrosis eventuating in chronic interstitial fibrosis and progressive chronic kidney disease (Figure 3) [38, 39]. 

This initial antigen presenting and T cell activation phase is followed by an integrative phase of the immune response [39, 40]. This subsequent effector phase is mediated by humoral factors released by the infiltrating and residual renal cells. The bidirectional cross-talk between the recruited infiltrating inflammatory cells and renal parenchyma, either by locally produced soluble cytokines or direct contact, ultimately modulates the course and severity of renal involvement (Figure 3) [6, 41]. In addition to potential mesenchymal transition (EMT), activation of the tubular epithelial cells and vascular endothelial cells up-regulate their expression of various cytokines that augment the role of the macrophages and fibroblasts [42]. In turn, the release of collagenases, elastases, and reactive oxygen species by the macrophages magnifies the injury initiated by the lymphocytes. Infiltrating neutrophils initiate the tubulitis observed in severe cases, whilst activated fibroblasts proliferate and alter the balance in favor of increased matrix synthesis rather than removal [6]. The varying signals that interact to modulate or amplify the inflammatory reaction of AIN are shown in Figure 3. 

The resultant activation of infiltrating cells and their interactions with renal parenchymal cells either suppress the effector phase, as in mild forms of AIN, or amplify it, as in severe forms of AIN. The duration and severity of the effector phase depends on when the antigenic source is eradicated if it is an infection, or discontinued if it is a drug. Ultimately, feedback mechanisms and removal of the inciting agent restore the inflammatory response to its baseline quiescent state with consequent recovery of normal kidney function in mild forms of injury or residual permanent fibrotic damage in its severe forms [6, 39, 40, 43]. 

Contrary to experimental data in support of anti-TBM disease in animal models of interstitial disease, anti-TBM antibodies are rarely detected in human AIN [6]. The same limitation applies for the role of activated B cells in eliciting an immune complex-mediated AIN. Cases in which fluorescent deposits of immunoglobulins were detected have been mainly in subjects with systemic diseases such as Sjögren’s syndrome or lupus, whose underlying autoimmune disease mechanism accounts for the deposition of immune complexes in the kidneys as well as other body organs. By contrast, the evidence in favor of cell-mediated disease as the cause of AIN is overwhelming. Available studies have not yet elucidated whether there is any difference in the diagnostic pattern of the infiltrating antigenically activated T cell subtypes. Reported differences may be due to individual genetic background, nature of the insult, and the point in time during the disease when biopsies were studied [6]. 

## Diagnosis 

As a clinical condition characterized by an acute onset of kidney injury, the principal differential diagnosis of AIN is its differentiation from ATN. The contrasting differentiating features of AIN from ATN are shown in Table 2. As a rule, the rapid onset of abnormal renal indices and oliguria favors ATN, whilst an insidious onset with a history of systemic symptoms (rash, fever, arthralgias, flank pain) favors AIN. 

### Structural 

The key structural differentiating feature between the two entities is the magnitude of interstitial edema and cellular infiltrates, which are more prominent in AIN, while the magnitude of tubular epithelial cell injury is more prominent in ATN [6]. Notably, the tubular injury of AIN (tubulitis) is a focal lesion with inflammatory cellular infiltrates penetrating and damaging the tubular basement membrane, with injury to the baso-lateral surface of adjacent epithelial cells. Tubulitis, considered as a reliable lesion for the diagnosis of acute renal allograft rejection, is also characteristic of severe AIN [44, 45]. By contrast, the tubular lesion of ATN is that of direct epithelial cell injury beginning with damage to the villi but a relatively well-preserved basement membrane, that is followed by increasing epithelial cell apoptotic or necrotic changes and sloughing into the lumen [46]. However, given the variable degree to which each of these features may be present in an individual case, and the overlap between the extent of edema and inflammatory cells it can be difficult to differentiate among them on morphologic features alone, at least in some cases [6]. 

### Clinical 

By contrast to infection-associated AIN, most cases of drug-induced AIN develop over several days, weeks, and months after exposure to the inciting agent. The classic clinical triad of low-grade fever (35 – 70%), fleeting skin rash (25 – 40%), and eosinophilia (35 – 60%) is not always present, and is certainly less common for all three to occur together. Their detection depends to some degree on the vigilance with which they are sought as they may be mild and transient. The full triad, more common (20%) with β-lactam antibiotics, may be present in less than 10% of other drug-induced AIN [46, 47, 48, 49]. Flank pain or a sense of fullness in the loins, reflecting edema-induced distention of the renal capsule, may be present in over 30% of cases when queried and can be the presenting symptom in some cases [50]. Gross hematuria may be present in 5 – 15% of drug-induced ATIN cases. A history of arthralgia may be elicited in more than 25% of cases [46, 47, 51]. 

### Laboratory 

**Figure 4.**
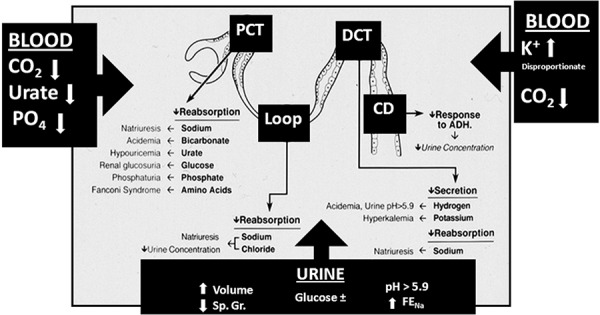
Schematic representation of the sites of tubular dysfunction in acute tubulointerstitial nephritis. The abnormalities in tubular handling of electrolytes are shown in bold lettering and their clinical manifestation in regular lettering. The boxed black arrows indicate the principal changes reflected in blood and urine tests. PCT = proximal convoluted tubule; DCT = distal convoluted tubule; Loop = loop of Henle; CD = collecting duct; Sp. Gr. = specific gravity; FE_Na_ = fractional excretion of sodium; PO_4_ = phosphate; CO_2_ = carbon dioxide content. (Reproduced with permission from reference number [6]).

Laboratory markers of tubular dysfunction are evident before decrements in filtration rate and consequent increments in blood urea nitrogen (BUN) and serum creatinine levels. The principal hallmarks of glomerular disease (salt retention, edema, hypertension) are characteristically absent. The early diagnosis of AIN by detecting tubular dysfunction (Figure 4) is central to its diagnosis at a potentially reversible stage [46, 47]. 

The impairment in kidney function varies, ranging from discrete selective abnormalities of tubular function to frank kidney failure, with or without oliguria [6, 46, 47, 48]. As a rule, increments in BUN and creatinine develop after tubular dysfunction is detectable and while the patient is still non-oliguric and actually polyuric. Oliguria develops if early features of AIN and evidence of tubular dysfunction go undetected and exposure to the offending agent continues. 

The pattern of tubular dysfunction that results varies depending on the major site of injury, whereas the extent of damage determines the severity of tubular dysfunction (Figure 4). Lesions principally affecting the proximal tubule result in bicarbonaturia (proximal renal tubular acidosis), glucosuria (renal glucosuria), aminoaciduria, β_2_-microglobinuria, phosphaturia, and uricosuria [6]. The presence and extent of these abnormalities can be determined by calculating their respective fractional excretions. A lower serum phosphorus or urate level in any azotemic patient should always suggest the possibility of AIN, as well as that of glucosuria on routine urinalysis when the blood sugar levels are normal. Lesions primarily affecting the distal tubule will result in a distal form of renal tubular acidosis, with hyperkalemia. Lesions that primarily affect the cortico-medullary junction disproportionately affect medullary structures essential to medullary hypertonicity and urine concentration resulting in variable degrees of nephrogenic diabetes insipidus, with persistent polyuria (non-oliguric acute renal failure), which almost always precedes the onset of oliguria in AIN [6]. These segmental considerations of tubular dysfunction notwithstanding, considerable overlap of varied tubular dysfunctions occur clinically as early warning hallmarks that presage the onset of renal failure. 

### Urinalysis 

Urinalysis is critical for early diagnosis (Table 2). Proteinuria, hematuria, and pyuria are present in most cases. The proteinuria is mild, seldom exceeds 2 g/day, and only rarely is in the nephrotic range, except in cases due to NSAIDs. Microscopic hematuria is present in 70 – 90% of cases; rarely, red blood cell casts may be detected. Pyuria is present in most cases [46, 47, 48]. 

The sterile pyuria of AIN is nonspecific except when eosinophils are detected in appropriately prepared and carefully examined urinary sediment [6, 46, 47]. The mere detection of eosinophiluria is not specific for AIN [6]. The sensitivity of eosinophiluria for the diagnosis of AIN has been estimated to be ~ 60% and its specificity to be 85%, with a positive predictive value of 38%. These figures, derived from retrospective chart reviews of clinically suspected, but not biopsy-proven, cases of AIN are at best guesstimates [52, 53, 54]. Eosinophiluria is present in ~ 15% of hospitalized patients due to a variety of other inflammatory diseases of the kidney and urinary tract (cystitis, prostatitis, obstruction, embolic disease, contrast dye), and is actually associated with AIN in only 14% of those with eosinophiluria [52]. Eosinophiluria is a better indicator of AIN when more than 5% of the urinary leukocytes are eosinophils in cases with significant pyuria [52]. White blood cell casts occur, and are fairly characteristic when they contain eosinophils [53]. Thus, the detection of eosinophiluria, although useful, is neither necessary nor sufficient for the diagnosis of AIN [54]. Its routine ordering in cases with no pyuria is a total waste. 

### Imaging studies 

Increased kidney size on ultrasonography, reflecting interstitial edema, is common but nondiagnostic of AIN. Radioactive gallium uptake by the kidney, reflecting interstitial cellular infiltration, can be present in one-third of cases, but lacks diagnostic specificity [55]. Unlike the pyramidal distribution of the lesions of pyelonephritis, those of AIN are more diffuse and reflect the disperse pathology described by Councilman (Figure 1). 

### Lymphocyte stimulation tests 

Apart from elucidating the immune-pathogenesis of AIN, the demonstration of circulating drug-reactive T cell clones in AIN patients provides a useful tool in determining the specific drug responsible for AIN [40, 41, 43]. Clinically, most patients with AIN are on more than one drug, and the empiric decision to stop all, or just the most suspicious drugs, leaves the specific causative agent undetermined. This is a matter of concern since drug-induced lymphocyte stimulation tests (DLST) reveal that the causative agent may not been suspected and sometimes the wrong drugs were removed, hence the risk of future re-exposure and its associated more serious renal injury [6]. The principal limitation of DLSTs in the diagnosis and treatment of AIN is limited availability, cost and time delay in performing them, but can be most useful where available [56]. Importantly, activated lymphocytes persist in the circulation for years after sensitization, and as such can be useful in identifying the causative agent even after recovery from AIN so that its future use can be avoided [41]. 

Relevant in this regard are studies in genomics with their potential of predicting the polymorphism that renders individuals susceptible to delayed hypersensitivity reactions that could allow for the choice or avoidance of drug classes in selected individuals [43]. 

### Biomarkers 

Using sophisticated technology (proteomics, gene arrays, imaging, etc.), major strides have been made in the past decade in identifying biological markers of AKI, with the possibility of its early detection and potential for improved management. Several prospective useful biomarkers have been identified, some of which have entered clinical practice (β1 and β2 microglobulins, MCP-1, NGAL, TGF-β, KIM-1, TIMP-2, IGFBP-7, L-type FABP), whilst many others are at various stages of investigation and development [57, 58, 59]. 

Concerning the utility of biomarkers in AIN, it should be noted that literally all biomarkers have been developed in subjects with a diagnosis of AKI, based on its definition as an increment of serum creatinine or a decrease in urine output. Clinically, their principal focus has been the differential diagnosis between pre-renal (potentially reversible) and intrinsic (potentially irreversible) acute renal failure, specifically that due to ATN [60]. In fact, the clinical applicability of most biomarkers is derived from initial studies in patients undergoing major vascular surgery in which there is an increased risk of ischemic tubular injury [59, 61]. They are then validated in epidemiologic studies of AKI, an over-encompassing term that does not differentiate increments in serum creatinine by etiology. Hence, the use of biomarkers in AKI currently has statistical rather than specific diagnostic utility [59, 62]. 

In the single available study of biomarkers in cases of biopsy proven drug-induced AIN, the average serum creatinine at the time of biopsy was ~ 2.5 mg/dL (202.56 ± 86.43 µmol/L); essentially the renal injury was well passed its initial stage of a tubulopathy into that of a drop in filtration rate [63]. In this study, increased levels of monocyte chemotactic protein-1 (MCP-1) were associated with interstitial edema and inflammatory cell infiltration, whereas neutrophil gelatinase-associated lipocatin (NGAL) showed the better correlation with tubular injury and atrophy. 

In sum, the applicability of biomarkers in the early diagnosis of AIN cases remains to be determined, while vigilance to the presence of tubular dysfunction in non-oliguric subjects remains a more useful diagnostic clue of AIN (Figure 4). 

## Causative agents 

Table 3 provides an attempt at the inclusive listing of the causative agents associated with AIN reported in the literature [6, 47, 64, 65, 66, 67, 68, 69, 70, 71, 72]. Currently, some 70% of reported AIN cases are ascribed to drug-induced interstitial nephritis. The number of drugs implicated continues to increase, as does that of the variation in their clinical and laboratory manifestations [43]. In the absence of a kidney biopsy, the association of AIN with most incriminated drugs is circumstantial. Even if a biopsy is performed, the results may be confounded by preexisting kidney disease, ischemic injury, or a nephrotoxic effect of co-administered drugs [6]. 

Apart from drugs and infections, AIN is well documented in systemic disorders and malignancies. Systemic disorders associated with AIN include autoimmune diseases [73]. A subtype of idiopathic AIN with a prominent lymphoplasmacytic infiltration of IgG4-positive plasma cells deserves highlighting due to its severity, increasing recognition, and favorable response to corticosteroids [74]. 

## Treatment 

Early diagnosis of AIN is the mainstay of its treatment. Underlying infections and associated systemic diseases should be treated appropriately and all suspected drugs discontinued. Unfortunately, in cases where systemic manifestations (fever, rash, arthralgia) are absent and laboratories abnormalities (eosinophilia, tubular dysfunctions) go unnoticed, and the diagnosis is considered only after the onset of azotemia or oliguria supportive measures, including dialysis, become necessary and the risk for permanent loss of kidney function increases. 

As a delayed hypersensitivity reaction, independent of the level of renal injury, the use of steroids is a question that arises in the care of AIN cases, but remains a controversial issue. Given the idiosyncratic nature of the disease, no controlled trials are available or likely feasible, and all available reports are retrospective in nature. The varied faces of the problem are illustrated in the following three studies of biopsy proven AIN reported in the past decade. In 2004, a retrospective report from a single center in the US with 60 cases of biopsy-proven AIN, 36 of whom were treated with steroids, found no statistical difference in their response compared to the 24 who were not treated [14]. By contrast, in a 2008 multi-center report from Spain of 61 biopsy-proven cases, the 52 patients treated with steroids showed a clear benefit in recovery of baseline function and discontinuation of dialysis, compared to the 9 who were not [75]. Importantly, better outcomes were achieved in those whose steroid therapy was initiated earlier (within 1 – 14 days) after diagnosis of AIN. In the third report from a single center in the UK of 49 cases, the 37 patients treated with steroids were less likely to require dialysis and achieved better recovery of kidney function than the 12 who were not; but unlike the report from Spain delay in initiating steroids made no difference [22]. 

Taken together, the bulk of the anecdotal literature and retrospective reports dating back to the 1960s indicates that steroids seem to exert a beneficial effect in AIN. A theoretical argument can be made that in available reports selection bias would favor those who are not treated with steroids, because it is patients who do not respond to discontinuation of the incriminated cause and sustain progressive loss of kidney function who are more likely to be treated with steroids. As such, steroids certainly deserve to be considered in patients with persistent kidney injury after the inciting agent has been discontinued, and in those whose biopsy reveals fibrosis, macrophage infiltration or granulomatous lesions that are associated with increased risk of permanent injury [6, 76]. The early initiation of steroids in idiopathic cases of AIN while conceptually appealing must be individualized, and based on consideration of its merits in each case. 

If steroids are used, a response usually is evident relatively early after initiation of treatment. The course of treatment should be brief, and steroids tapered and discontinued if no response is observed after 4 weeks of therapy [6]. If kidney function improves, steroid therapy should probably be maintained and slowly tapered once a stable baseline kidney function level is attained. In cases of rapidly deteriorating kidney function consideration should be given to pulsing with steroids prior to starting maintenance therapy. Whenever steroids are used, their potential benefit should be weighed against the risk of a co-existent infection as the cause of AIN. For patients who fail to respond to steroids, become steroid dependent, or are intolerant of them a beneficial effect has been reported with mycophenolate mofetil [77, 78]. 

In TINU, both the ocular and renal changes respond to a brief course of steroid, but the disease can relapse [79, 80, 81]. The uveitis may be asymptomatic indicating the need for ophthalmologic examination in cases of idiopathic AIN [80]. In children and adolescents, the long-term prognosis is good, with recovery of kidney function and no visual loss. Spontaneous remission without steroid treatment has been reported in children; hence the reluctance to use steroids in this age group [81]. 

### Prognosis 

Permanent kidney injury secondary to AIN is more likely to occur in older patients and is more severe in those who become oliguric, develop azotemia, and require dialysis [6]. Dialysis may be required in ~ 1/3 of these patients. Reversal of kidney injury and return to baseline kidney function is the rule in 60 – 65% of cases. Irreversible kidney injury can occur but is rare (~ 5% to 10%), while partial recovery with persistent impairment of kidney function is relatively more common (10 – 20%), especially in cases where interstitial fibrosis and granulomas are present in biopsy specimens and those with pre-existing chronic kidney disease [46, 48]. 

## Conclusion 

AIN is an under recognized cause of AKI. The key to its early detection is vigilance to the onset of tubular dysfunction in non-oliguric individuals, especially in the presence of gradual reduction in reported eGFR. The majority (70%) of reported cases are due to drug-induced delayed-hypersensitivity. The primary treatment is identification and withdrawal of the offending agent. A short course of corticosteroids deserves consideration if a drug withdrawal fails. 

## Conflict of interest 

None. 

## Financial disclosures 

None. 

**Table 1.** Incidence of AIN in published kidney biopsy registries*.

Year (ref)	Period	M/S*	Country	Biopsies (number)	Age	ARF cases	AIN cases	% AIN of ARF	% AIN of total
1988 [9]	1970 – 1986	S	UK	2,600	N/A	N/A	51	N/A	1.9
1997 [10]	1987 – 1993	M	Italy	10,357	N/A	952	104	11.3	1.0
1998 [11]	1978 – 1998	M	UK	7,161	N/A	1172	76	6.5	1.1
2000 [12]	1987 – 1999	M	Saudi Arabia	1,013	N/A	N/A	99	N/A	9.8
2004 [13]	1979 – 2002	M	China	13,519	N/A	N/A	202	N/A	1.5
2004 [14]	1988 – 2001	S	USA	2,598	65	583	60	10.3	2.4
2004 [15]	1994 – 2000	M	Czech	4,004	39	N/A	176	N/A	4.4
2006 [16]	1986 – 2002	S	India	5,415	N/A	N/A	135	N/A	2.5
2006 [17]	1995 – 2004	M	Romania	635	70	76	9	12	1.5
2007 [18]	1977 – 2005	S	Italy	3,269	42	N/A	137	N/A	4.2
2009 [19]	1987 – 2006	M	Serbia	1,626	39	N/A	16	N/A	1.0
2010 [20]	1998 – 2007	S	Iran	1,407	37	79	28	35	2.0
2011 [21]	2000 – 2009	S	South Africa	1,284	38	269	18	6.7	1.4
2012 [22]	1999 – 2008	S	UK	1,037	64.4	N/A	49	N/A	4.7
2013 [23]	2009 – 2010	M	Japan	7,442	47	N/A	112	N/A	1.5
2013 [24]	1994 – 2009	M	Spain	14,190	63	3059	383	12.9%	2.7
2014 [25]	2000 – 2010	S	USA	3,765	41	N/A	150	N/A	4.0

*M/S: M (multiple centers), S (single center). Not all data available for analysis. Criteria for selection: 1) English-language publication; 2) Registry contained > 500 kidney biopsies; 3) Prevalence of AIN was clearly mentioned. Search conducted on PubMed using the following key words: kidney biopsy, interstitial nephritis, tubulointerstitial nephritis, acute, registry, database, glomerulonephritis. In many registries, the %ARF is not reported.

**Table 2.** Distinguishing features of ATN from AIN.

	Acute Tubular Necrosis	Acute Interstitial Nephritis
Onset following injury	Hours to days	Days to weeks
Urine volume	Oliguria < 500 mL/d	Polyuria (> 2,000 mL/d)
Clinical features	Hemodynamic instability	Rash (25 – 40%), fever (35 – 70%), back pain (25 – 40%), arthralgia (25 – 40%)
Histology	Tubular epithelial cell injury	Interstitial cellular infiltrates, edema, tubulitis
Eosinophilia	Absent	Present (35 – 60%)
Tubular dysfunction^†^	Rare	Very common
FE_Na_^#^	> 1%	> 1%
Urine microscopy	Epithelial cell and broad granular casts	Hematuria (70 – 90%), pyuria (75 – 85%), eosinophiliuria* (variable)
Treatment	Hemodynamic resuscitation, withdrawal of nephrotoxic agent, supportive care	Withdrawal of offending agent, supportive care, limited trial of steroids
Prognosis	Recovery (65%), CKD (~ 35%)	Recovery (65%), CKD (~ 35%)

^†^See Figure 4 for detail; ^#^fractional excretion of sodium; *assumes more than 5% of urinary leukocytes are eosinophils.

**Table 3.** Published causes of AIN.

Classes	Specific Agents
Drug-induced: antibiotics	Incidence > 50 cases reported Methicillin, rifampin, ciprofloxacin Incidence < 30 cases reported Acyclovir, amoxicillin, ampicillin, atazanivir, carbenicillin, cefazolin, cefaclor, cefamandole, cefotaxime, cefotetan, cefoperazone, cefoxitin, ceftriaxone, cephalothin, cephalexin, cephradine, cephaloridine, cephapirin, chloramphenicol, clindamycin, dimethoxyphenylpenicilloyl, doxycycline, erythromycin, ethambutol, flurithromycin, indinavir, levofloxacin, lincomycin, linezolid, mezlocillin, minocycline, moxifloxacin, nafcillin, netilimicin, nitrofurantoin, norfloxacin, oxacillin, penicillin, piparcillin/tazobactam, polymyxin,trimethoprin-sulfamethaxozaole, tetracycline, telithromycin, vancomycin
Drug-induced: non-steroidal anti-inflammatory drugs (NSAIDs)	Incidence > 30 cases reported Non-selective COX inhibitors: fenoprofen > ibuprofen > naproxen; COX-2 inhibitors: celecoxib > rofecoxib Other agents: aceclofenac, indomethacin, diclofenac, diflusinal, flurbiprofen, mefenamate, phenazone, phenylbutazone, noramidopyrine, nimesulide, prioxicam, sulindac, tolmentin, zomepirac
Drug-induced: proton-pump inhibitors & H_2_-Antagonists	Incidence > 50 cases reported Omeprazole Other PPIs: lansoprazole > pantoprazole > esmoprazole > rabeprazole; H2 Antagonists: cimetidine, famotidine, ranitidine
Drug-induced: anti-neoplastic agents	Incidence >20 cases reported Ifosfamide Other agents: adriamycin, BCG, bevacizumab, carboplatin, cediranib, gemcitabine, ipilimumab, interferon, lenolidomide, pemetrexed, sorafenib, sunitinib
Drug-induced: diuretics	Incidence < 10 cases reported Acetazolamide, chlorthalidone, ethacrynic acid, furosemide, hydrochlorothiazide, indapamide, tienilic acid, triamterene
Other drugs	Incidence >10 cases reported Allopurinol, 5-Aminosalicylic acid Others: acetaminophen, anisoindione, aminopyrine, amidopyrine, amphetamine, aristolochic acid, armodafinil, aspirin, azathioprine, captopril, carbamezapine, clofibrate, clozipine, certirizine, cinitapride, clomipramine, cocaine, creatine monohydrate, deferasirox, diltiazem, diphenadione, ergotamine, etanercept, exenatide, fluindione, foscarnet, hydroxyethyl starch, interleukin, isotretinoin, immunoglobulin, griseofulvin, kudzu root juice, levetiracetam, liraglutide, methyldopa, paraphenylene diamine, phenazopyridine, phendimetrazine, phenobarbitol, phenteramine, phenytoin, piperazine, propylthiouracil, quinine, rosiglitazone, rosuvastatin, sirolimus, sulfinpyrazone, valproate,varenicline, warfarin
Infections	Adenovirus, ascaris, babesiosis, candidia, coxiella, cryptococcus, cytomegalovirus, diptheria, epstein-barr virus, hantavirus, hepatitis a, hiv, histoplasmosis, hydatid, influenza a, leptospirosis, legionella, microsporidia, mycobacteria, mycoplasma, polyoma virus, pyelonephritis (multiple organisms), rickettsiae, salmonella, streptococcus, toxoplasma, yersinia
Metabolic	Calcium, heavy metals (e.g., mercury, lead), oxalate, urate
Autoimmune	Incidence > 50 cases reported Systemic lupus erythematosus Incidence < 20 cases reported ANCA vasculitis, autoimmune pancreatitis, glomerular disease, inflammatory bowel disease, mixed cryoglobulinemia, primary biliary cirrhosis, polyarteritis nodosa, polymyositis, sarcoidoisis, Sjögren’s syndrome
Malignancies	Leukemia, lymphoma, multiple myeloma
Other	Anti-TBM, idiopathic, IgG4 disease, insect bites, rejection of transplanted organ, snake bite, TINU syndrome

The list was compiled from PubMed search using the terms “‘Acute Interstitial Nephritis” and “Acute Tubulointerstitial Nephritis”. The search includes all listed publications (4840) prior to March 31, 2014.
